# Fumonisin Intake from Consumption of Wheat- and Corn-Based Products in Hungary

**DOI:** 10.3390/toxins17120566

**Published:** 2025-11-22

**Authors:** Tamás Schieszl, Ákos Jozwiak, Miklós Süth, Imre Nemes, Melinda Kovács, Andrea Zentai

**Affiliations:** 1 HUN-REN-MATE Mycotoxins in the Food Chain Research Group, Guba S. Street 40., 7400 Kaposvár, Hungary; 2 MATE Doctoral School of Animal Science, Guba S. Street 40., 7400 Kaposvár, Hungary; 3 Department of Digital Food Science, Institute of Food Chain Science, University of Veterinary Medicine Budapest, István Street 2., 1078 Budapest, Hungary; 4 Institute of Food Chain Science, University of Veterinary Medicine Budapest, István Street 2., 1078 Budapest, Hungary; 5 External Department of Food Chain Safety (NÉBIH), Institute of Food Chain Science, University of Veterinary Medicine Budapest, István Street 2., 1078 Budapest, Hungary; 6 Agrobiotechnology and Precision Breeding for Food Security National Laboratory, Department of Animal Physiology and Health, Institute of Physiology and Animal Nutrition, Hungarian University of Agriculture and Life Sciences, Guba S. Street 40., 7400 Kaposvár, Hungary

**Keywords:** fumonisins, FB1, FB2, mycotoxins, exposure, dietary

## Abstract

Fumonisins are mycotoxins commonly found on corn and other grains, and have been linked to several health concerns. The aim of this study is to estimate the exposure of the Hungarian population to fumonisins from regular food consumption. Fumonisin B1 and B2 concentrations were determined in commercially available corn-, wheat- and rice-based foods. Daily intakes on an individual level were calculated deterministically based on recent individual food consumption data distributions, and the average contamination of the concerned food categories. The most significant sources of fumonisins were corn flour, cornmeal, cornflakes, other corn-based products, and wheat-based products (average total fumonisin contents in the middle bound scenario were 0.115, 0.074, 0.052, 0.091 and 0.077 mg/kg, respectively). In cases where the concentration values measured below the analytical determination limits, these were substituted by half of the corresponding limits (middle bound scenario). Mean and high (95th percentile) exposures to fumonisins B1 + B2 were 0.101 and 0.258 µg/bw kg/day for adults, and 0.313 and 0.744 µg/bw kg/day for children, respectively. Our results show that about 1.5% of children’s exposure could exceed the tolerable daily intake limit (TDI) of fumonisins, established by EFSA in 2018, meaning that potential health risks cannot be ruled out for a low proportion of consumers. We found that besides corn-based products, wheat-based food products could also contribute to total intake, due to their high consumption levels. Constant monitoring of fumonisin levels in corn- and wheat-based products is recommended to safeguard consumer health.

## 1. Introduction

Mycotoxins are secondary metabolites of molds that can occur in the food chain all around the world. Toxin contamination of the crops can take place both during storage and/or on the field [1]. The level of contamination depends on several environmental factors, and also on the interaction between mold species and plant cultivars [2,3,4]. Climate change may affect fungal distribution, plant resilience, host–pathogen interactions and toxin production. In Europe, the changes favour the spread of many mycotoxin-producing fungal species [3].

The type and concentration of mycotoxins in the food chain can show huge variance. Industrial and household processes (such as peeling, sieving or any other type of separation) are also able to affect the toxin levels of foods consumed [5,6,7,8]. Despite treatments involving heat, such as baking or cooking, mycotoxin contamination can still persist [9].

Mycotoxins can have a variety of toxic effects on humans and animals, such as carcinogenic, hepatotoxic, immunosuppressive, nephrotoxic, teratogenic, and mutagenic effects, as well as reproductive toxicity or even acute intoxication [10]. Fumonisins specifically are inhibitors of ceramide synthase, which leads to the intracellular accumulation of free sphinganine (and to a lesser extent sphingosine), and disturbance of the lipid composition of the cell membrane. FB1 exerts a further disturbing effect on the fatty acid profile via the induction of oxidative stress [11]. To protect people, concentrations of these toxins need to be kept as low as possible [12].

In order to minimize the harmful effects of mycotoxins on the public, it is crucial to make comprehensive strategies that include improved detection methods, extensive research and customized management strategies [9]. Regular monitoring of foods and feeds is also imperative in order to lower the health risks [10,13].

Fumonisins are a group of mycotoxins produced by certain *Fusarium* species, primarily *Fusarium verticillioides* and *Fusarium proliferatum*. Fumonisins commonly occur on corn and other grains [14]. The most toxic and common in food is fumonisin B1 (FB1) [14]. In the Central European context, contamination rates were 55 and 60% in 2024 and the first half of 2025, respectively [15,16]. Fumonisins have been related to developmental disorders, to liver and kidney toxicity, and to liver and esophageal cancer [10,12].

Food safety authorities set limit values in order to protect the population from exposure at harmful levels. These limits are based on scientific experiments, toxicological studies and hazard analysis (hazard identification and characterisation) [17,18,19,20]. In addition, mitigation strategies could also be directed at decreasing exposure. For instance, recent research suggests that natural dietary additives with antioxidant and gut-protective properties may help mitigate toxicity by enhancing intestinal barrier function and reducing oxidative stress [21]. For example, in the context of mycotoxin-induced oxidative and mitochondrial damage, rooibos (*Aspalathus linearis*) has been proposed as a protective agent: preclinical and human studies show it can reduce lipid peroxidation markers (e.g., TBARS, CDs), modulate glutathione redox balance, and thus potentially buffer toxin-induced oxidative stress [22,23].

The CONTAM Panel of European Food Safety Authority (EFSA) decided to define tolerable daily intake (TDI) not just for one toxin, but a group of toxins which show structural and toxic potential similarities. For this, 1 µg/kg body weight per day (µg/kg bw day) was established as a group TDI for fumonisin B1 (FB1), fumonisin B2 (FB2), fumonisin B3 (FB3) and fumonisin B4 (FB4), based on increased incidence of megalocytic hepatocytes found in a chronic study with mice [24].

In order to perform risk characterisation, outcomes of hazard characterisation and exposure assessment are needed. Exposure assessment can be performed by using food consumption data and food contamination data. It is also possible to use biomarkers such as urine or blood. Both methods require the detection of harmful substances [25,26].

Liquid chromatography mass spectrometry (LC-MS) is precise and commonly used for determining mycotoxins. Widely used procedures are based on electrospray ionisation (ESI) and a quadrupole mass analyser. Immunochemical bead tests, such as enzyme-linked immunosorbent assay (ELISA), are also available on the market. These methods show high sensitivity, but are only capable of measuring a few mycotoxins at the same time [13,24,27,28].

In our previous study, we estimated fumonisin dietary exposures in Hungary based on measurements in commercially available food raw materials. The estimated intakes remained below the TDI [29]. Since the completion of that study, additional data have become available, which may allow for a more detailed assessment of consumer exposure. The novelty of the present study lies in the fact that, for the first time, contamination data could be compared with temporally relevant consumption data from the 2018–2020 EFSA survey. This represents a significant improvement over our previous study, where contamination data from 2017 to 2019 could only be assessed against outdated consumption figures from 2008, due to the lack of more recent data at the time. The use of up-to-date consumption data enables more accurate exposure assessments and thereby allows for more reliable conclusions regarding potential health risks to the population. Therefore, the aim of the present study is to estimate the exposure of the Hungarian population to fumonisins from commercially available corn-, wheat- and rice-based foods that are particularly important from the point of view of *Fusarium* toxins, considering recent national food consumption data, and to compare the obtained data with the TDI value established by EFSA. These results will be of importance in the Central European context, providing the most updated information relevant to consumer risk in this region.

## 2. Results

The measured concentrations of FB1 and FB2 in different food groups—numbers of samples below and above the limit of detection (LOD) and limit of quantification (LOQ), means, medians and maximums—are shown in Table 1.

The prevalence of FB1 was higher; it was found in higher concentrations than FB2 (Table 1). It can be observed that more concentrations were measured above the LOQ in corn products (17/60 corn flour samples, 14/73 other corn-based products) than in other food categories. However, the wheat-based product category is also significant (15.3% positivity), and was more contaminated with FB1 and FB2 than the other wheat product categories (i.e., fine wheat flour or whole wheat flour). Considering rice-based products, while the frequency of fumonisin occurrence was low (one positive sample among brown-, white- and rice-based products each), the contamination level was considerable (FB1 levels of 0.388 mg/kg, 0.557 mg/kg and 0.144 mg/kg, respectively). FB2 exceeded the LOQ in only 1–2 cases; however, if we examine the whole 2017–2023 period together (including those results which were published previously, Table A1), its occurrence in corn products was more significant (24.6%).

Summed FB1 and FB2 contents of different food groups (using lower bound, middle bound and upper bound calculations of LB, MB and UBis) are shown in Table 2. More detailed percentile data are contained in Table A2 and detailed results for the total 2017–2023 time interval are reported in Table A3.

The most significant sources of fumonisins were corn flour, cornmeal, cornflakes, other corn-based products, and wheat-based products (average total fumonisin contents in the MB scenario were 0.115, 0.074, 0.052, 0.091 and 0.077 mg/kg, respectively). The high proportion of corn products among the sources of these toxins is noteworthy. However, the highest concentration (0.832 mg/kg) was found in white rice; however, that was only a single outlier, as most of the rice samples did not contain any measurable fumonisins. With regard to all the results measured from the beginning of our project, the highest concentration for FB1 + FB2 (2.371 mg/kg) was found in cornmeal (Table A3).

Comparing the measured concentrations with the maximum limits set by Commission Regulation (EU) 2023/915, all the values were compliant. However, considering all the samples measured from 2017, one corn flour sample (2.194 mg/kg), two cornmeal samples (2.371 mg/kg and 1.489 mg/kg), and one sample of the other corn-based, snack-like products (1.1 mg/kg) had total fumonisin contents above the regulatory limit.

The results of the exposure estimation of total FB1 + FB2 are shown in Table 3. Results for the total 2017–2023 timespan are reported in Table A4. No significant differences were found between the exposure results based on the two time periods.

Using the LB estimation, the TDI (1 µg/bw kg/day) was not reached in the group of adults. The highest calculated intake was about one third of the TDI. Using the UB estimation, which represents a worst scenario with regard to the undetected results, we found ~0.2% of adults and 11.3% of children exceeding TDI. Children’s exposure was two to three times higher than adults based on median, mean, and 90th percentile values. Using the MB estimation (which we think is the most realistic), the calculated exposures were found to not reach even 75% of the TDI in the group of adults. The mean exposure of adults was 0.101 µg/bw kg/day, while high (P95) exposure was 0.258 µg/bw kg/day. With the same method, mean exposure of children was 0.313 µg/bw kg/day, and intake at the 95th percentile was 0.744 µg/bw kg/day. The TDI was exceeded in ~1.5% of children’s exposures.

**Figure 1:**
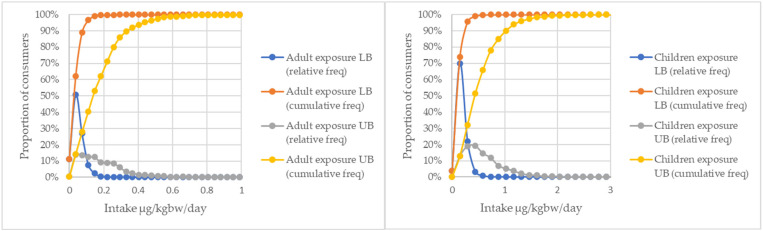
Relative and cumulative frequencies of total fumonisin intakes in adults and children in LB and UB, respectively.

Figure 1 illustrates the distributions of calculated exposures for adults and children, regarding both their relative and cumulative probabilities. The cumulative frequency curves show the proportion of consumer intakes falling below the specified levels, allowing us to calculate the ratio of consumers exceeding a given intake. The curves present the distributions from the LB and UB outcomes, while also demonstrating the difference in results between the two scenarios.

The contributions of each food category to the total intake of fumonisins at different percentiles are presented in Table 4 for both adult and child consumers. The three food categories that contributed the most to the intake were wheat-based products, fine wheat flour and white rice. Although there were differences depending on the age group and percentile, it was clearly shown that wheat-based food categories contributed the most to intake. Given that fumonisins were found in higher amounts in corn-based products (mean concentrations were greater in corn flour, cornmeal and other corn-based products than in wheat-based products), this result draws attention to the role of consumption values contributing to the intake of contaminants. This is also noteworthy because there are regulations regarding maximum levels only for corn and corn-based food products, and other food ingredients are not regulated [30].

## 3. Discussion

The results of our exposure calculations highlight the possibility that the tolerable daily intake of fumonisins could be reached by the usual consumption of wheat- and corn-based foods. Considering the MB scenario, which calculates with half the determination limit in cases of results below the LOD, we found that a small fraction (~1.5%) of children’s exposure could exceed the toxicological reference value. The higher exposures calculated for the younger generation is not an unusual fact, as their relatively lower body weight leads to relatively higher intakes on a body weight basis. According to EFSA [24] and WHO/JECFA [19] evaluations, children may have higher exposure levels to fumonisins due to their higher food consumption per body weight and their developing physiological systems, which justify specific attention in risk assessment frameworks. Moreover, children may be particularly susceptible to toxicity because of their hormonal and developmental sensitivity, especially in relation to oxidative stress-mediated endocrine disruption [31]. This underlines the fact that special consideration is needed for children because of their vulnerability.

It is worth noting that, beyond fumonisin-related risks, a broader body of research suggests that environmental or xenobiotic exposures may contribute to immune dysregulation and downstream health complications, underscoring the complexity of systemic effects in exposure assessment [32].

Wheat is one of the most significant human food sources [33]. We also found that an average person consumes the highest quantity from the food category of wheat-based products. Based on all registered consumptions, the mean consumptions of corn-based products, canned corn, cornmeal and corn flour were 1.61 (224 data), 1.78 (196 data), 1.18 (6 data) and 0.48 g/kg bw (7 data), respectively, while the mean consumption of wheat-based products was 2.30 g/kg bw (2274 data). We cannot ignore the high proportion of contamination in corn products; however, the contamination of wheat-based products (although their lesser contamination compared to corn) is also cause for concern due to the high consumption of this category. Li et al. [34] found much higher FB1 contamination in corn (98% prevalence) than in wheat (6.2% prevalence) in China. In Serbia, like us, Stanković et al. [35] found significant levels of FB1 contamination in wheat (92% prevalence, 750–4900 μg/kg FB1) in 2007.

The three food categories that contributed the most to the intake in our calculations were wheat-based products, white rice and fine wheat flour. While there were some differences according to the percentile intake and age group, it seems clear that wheat and rice could contribute to the intake of total fumonisins. While corn-based products, particularly corn flour, cornmeal and other corn-based products, had the highest mean concentrations, consumption of wheat-based products is considerable in Hungary and could lead to considerable exposure to fumonisins. Regarding the role of rice in fumonisin exposure, we must note that the overall contamination was not outstanding in our samples, and an explanation of our finding lies in the methodology; while only one sample was contaminated above the limit of determination, it was contaminated by both FB1 and FB2, which led to a high total concentration. Combining such a high level with a considerable consumption of different rice-based products could result in higher exposure values. Therefore, we must note that occasional contamination spots and the corresponding consumption of rice, which is noteworthy in our region, could lead to relevant exposures in sporadic cases. However, considering the frequency of fumonisin detection in our rice-based products, we conclude that the real risks would be negligible.

What also needs to be added is that our food category of wheat-based products contained different types of products including bakery products, pasta or muesli. These are heterogenous in their ingredients, which adds much uncertainty to the linking of concentration and consumption data as a group. Disaggregating this food category into similar subgroups would decrease much of this uncertainty, which we could not do in our case due to the low number of samples.

Further measurements and calculations on samples with clearly defined contents would provide more valuable insights into the relevance of wheat-based product consumption. It would also be interesting to investigate the contribution of fiber-enriched whole grain products to overall consumption and fumonisin intake, as well as the potential differences between organic and conventional products, given the current consumer trends driven by environmental and health motivations.

A limitation of our calculations is the relatively high number of results below the determination limit of the analytical method. While the low frequency of contamination is favourable, it provides a methodological challenge to model non-detection. The substitution of these values with the lowest and highest possible values (lower and upper bound scenarios) could lead to different conclusions in our case, because the estimated risk was negligible with the former, while it was considerably higher in the latter, particularly in the case of children.

We therefore included a middle bound scenario, substituting non-detection by half the determination limits. We consider this option the most realistic one. Results below the LOD mean show that the measurement is not reliable at such low concentrations. Therefore, it is possible that the real value is zero, but it is definitely below the LOD value. We do not know for sure where—in the range between zero and the LOD—the true value lies. Therefore, we consider half of the LOD to be the most likely estimate.

In addition, the number of samples per food category may be limiting in giving an extensive overview of the real contamination situation. We aimed to mitigate this effect by the inclusion of all the concentrations measured during the lifespan of our study, which provides a more balanced view of the general contamination level. However, we found no remarkable differences between the contamination and estimated exposure levels between the two time periods.

Our findings consolidate previous research results stating that fumonisins are now widely present in Central Europe. In the Central European region, fusarium toxins (deoxynivalenol, zearalenone and fumonisins) are the most prevalent mycotoxins. (In the biggest part of the world, these compounds are also among the most highly prevalent toxins.) For example, in the year 2023, 77% of European corn samples were contaminated with deoxynivalenol (DON) and fumonisins (FUM), followed by zearalenone (ZEN) (66% prevalence). DON and FUM have also the highest average, median and maximum concentrations [36,37,38]. In 2024, contamination rates for DON, FUM and ZEN were 63, 61 and 60%, respectively, in Europe and 76, 55 and 58% in Central Europe, while in the first 6 months of 2025, these levels were 85, 60 and 71% in Central Europe [15,16]. In a Hungarian context, scarce data have been published on fumonisin occurrence. Fumonisin B1 was frequently detected in maize-based foods in the first part of our study (e.g., ~51.6% of maize flour and ~41.9% of maize grit samples), with average concentrations in the ranges of 0.13–0.20 mg/kg (FB1) and 0.03–0.10 mg/kg (FB2) in contaminated samples. Although a few samples slightly exceeded EU regulatory limits, the estimated mean and high percentile dietary intakes remained below the TDI of 1 µg/kg bw/day, indicating low risk under current exposure conditions [29]. In addition, fumonisins were measured in the urine of 41 healthy and 19 coeliac patients in 2021. Creatinine-normalised mean FB1 concentrations were 0.413 ng/mg and 0.341 ng/mg in the urine of healthy and coeliac patients, respectively. Estimated mean probable daily intakes for healthy and coeliac patients were 0.225 and 0.215 µg/kg bw/day based on piglet excretion data, and 1.172 and 1.116 µg/kg bw/day based on human excretion data, respectively [39].

In a broader context, previous European surveys generally showed no or very low risk for fumonisin. In the first French total diet study [40], the 95th percentile exposure was between 12 and 0.29% of TDI and none of the individuals’ theoretical intake exceeded the TDI. This was confirmed in a second study [41], in which FB1 and FB2 were present in bread, dried bread products and breakfast cereals; however, the risk of exposure was considered to not be a concern for the general population.

In a total diet study in the Netherlands, the exposure to FB1 + FB2 + FB3 the P95th percentile exposure in UB scenario was max. 13.8% (<6 years of age) and 24% (>7 years of age) of TDI. Intake of the grain-based products contributed the most to dietary exposure [42].

In an Italian study, the exposure of celiac patients to the main maize-contaminating mycotoxins was evaluated, based on the occurrence data obtained and referring to the food consumption survey published by the Italian Institute for Nutrition [18]. The highest exposure to fumonisin in the worst case (P95 and UB scenario) was 1385 ng/kg bw/day in children under 10 years old; however, in none of the individual cases was the TDI value exceeded.

In contrast to several previous studies, our findings indicate that dietary fumonisin exposure in Central Europe could exceed the TDI, which may require the constant attention of authorities to protect consumers.

Biomonitoring data also support the real risk of human fumonisin exposure. While direct comparisons of these data with our results are difficult due to the different methodologies, our results add further evidence to previous conclusions derived from these studies regarding human exposure. In a study covering the period 2010–2017, urinary biomarker data from nine European countries were used for modelling exposure. In the case of fumonisins, the estimated mean probable daily intake (PDI) value was about nine times higher than the TDI value [43].

On the other hand, the mean PDI for fumonisins was below the TDI and the PDI did not exceed the TDI in any case in a study of human urinary mycotoxin excretion patterns [44].

A review regarding mycotoxin biomarkers in human urine samples was undertaken based on relevant studies published between 2009 and 2018; in total, this included 56 articles representing four continents (Europe, Asia, Africa and America). Twenty-four studies from Europe were included. Data indicated high rates of dietary exposure to fumonisin, especially in African and Asian countries showing worrying levels for FB1. FB1 was frequently reported in Europe as well, with considerably lower incidence and calculated PDI values [45].

The results of the biomonitoring studies cannot be compared, due to the often significant differences in individual methodological elements (e.g., criteria for selecting volunteers, method of urine sample collection, using different excretion rates). However, they indicate that, although to varying degrees, human exposure to fumonisins is a real problem.

These studies from across different continents in the world also demonstrate the high variability of fumonisin occurrence, which depends on several factors including climate. Environmental conditions such as temperature, humidity, and water stress are known to influence *Fusarium* growth and fumonisin production; moreover, agronomic practices like fertilizer application rates, insect control, sowing date, tillage and residue management have been shown to modulate fumonisin contamination substantially [46,47,48]. Different food consumption habits also contribute to the differences. The more favorable situation in Europe may be partly due to the very strict regulation and risk management of the European Union. On the other hand, our findings and other European results also highlight the possibility that high fumonisin contents can be found in cereal products within Europe as well, just like in any other continent. Our results, with occasional high fumonisin contents in certain commercial food products, are in line with those published in a European context, and stress the need of constant monitoring of fumonisins in corn- and wheat-based products.

This mycotoxin exposure assessment conducted in Hungary holds substantial relevance for the Central European region. This is due to shared dietary patterns, similar agro-climatic conditions, and comparable agricultural and storage practices across neighboring countries. Moreover, the lack of comprehensive, population-based exposure data in the region further enhances the value of such a study. Hungarian data can thus serve as a reference for broader regional risk assessments and may inform food safety policies. Our focus was set on fumonisins, for which more data regarding exposure and hazard is needed [49]. Finally, climate change may alter the mycotoxin profile of a region [50]. This is why continuous monitoring is very important, especially for vulnerable populations such as children.

## 4. Conclusions

The estimated fumonisin exposure of Hungarian consumers from corn- and wheat-based products could reach the TDI set by EFSA in a proportion of cases. Our dietary exposure assessment applying fumonisin concentration data measured in popular food products and their consumption among Hungarian consumers suggests that about 1.5% of children consumers could be exposed to quantities higher than the toxicological reference value, considering the middle bound scenario in cases of non-detected results. Acknowledging the uncertainties of our calculations (using aggregated product categories and the relatively high number of measurement results below the LOD), it is important to note the contribution of wheat-based products, particularly in light of the cumulative consumption of these products. For future studies, it is desirable to measure fumonisin content in popular food products, as the quantity of consumption could drive relevant risks in spite of relatively lower contamination levels. The current trends of fiber-enriched whole grain product consumption and organic products are also an important research direction in terms of fumonisin exposure in the future.

Finally, the high concentrations found in corn-based products stress the important contribution of corn to overall fumonisin exposure and underline the need to maintain continuous monitoring of contamination in these food categories.

## 5. Materials and Methods

### 5.1. Collection of Food Samples and Measurement of Mycotoxin Contamination

Corn, rice and wheat were selected as the main ingredients in our study based on literature data on fumonisin occurrence, and also on their popularity within Central European consumers. We collected corn-, wheat- and rice-based products from the Hungarian market in three metropolitan regions and their surrounding areas, i.e., Kaposvár, Budapest, and other cities, e.g., Debrecen, Keszthely, Székesfehérvár and Solymár. The samples were purchased in grocery stores, large grocery chains (Auchan, Spar, Coop, Lidl, etc.) and smaller stores, which sell organic products. A total amount of 1004 samples was purchased in 6 years (from August 2017 to November 2023), including corn flour, cornmeal, corn flakes, canned corn, and other corn-based, snack-like products (extruded corn bread, tortilla chips, popcorn, nacho, maize chips, etc.), wheat flour, whole wheat flour and other wheat-based products, in addition to white rice, brown rice and rice-based products. Samples were randomly selected, collecting as many minor and leading brands available on the market as possible. All information about the samples (i.e., producer, country of origin and distributor) was obtained from the products’ labels and recorded. The samples taken in 2017–2018 were analysed in a separate publication [29]. Considering this and the slight modification in the sampling plan during the project (i.e., we included wheat products in the sampling plan in 2019), the present study focuses on the samples taken between 2019 and 2023 (*n* = 677). A summary of the analysed products is provided in Table 1 and A1 summarizes details of all the products collected through the whole timeframe of our project (2017–2023).

We determined FB1 and FB2 concentrations in the samples by using high-pressure liquid chromatography–mass spectrometry (LC-MS). As the samples arrived at the lab, we provided them an identification number, and then recorded their main characteristics. Afterwards, they were photographed and then lyophilized (Christ Alpha 1-4 LSC plus, Osterode am Harz, Germany). If the sample was not a powder, it was ground (ETA Vital Blend II blender; ETA a.s., Praha, Czech Republic). An amount of 5 g of sample was measured in 50 mL centrifuge tubes and 20 mL of extractant (acetonitrile: water = 1:1 + 0.1% acetic acid) was added. This was shaken for about 30 s on a test tube shaker (Velp; Lelystad, The Netherlands). The resulting mixture was shaken for 15 min in an ultrasonic bath (Elma Transsonic T 460; Amora, Portugal), because this helps to dissolve mycotoxins, and then for another 60 min on a circular shaker (Edmund Bühler; Dual-Action Shaker KL2; Bodelshausen, Germany) at 420 rpm to dissolve the mycotoxins. After that, the sample was first centrifuged for 5 min at 4000 rpm (Heinz Janetzki KG; T23, GDR: Liebzig, Germany), then centrifuged for 10 min at 14,000 rpm (VWR MEGA STAR 600R, Leuven, Belgium). One milliliter of the supernatant was measured using a Shimadzu LC/MS 2020 (Kyoto, Japan). The temperature of the column (Cognet Diamond Hydride—4 µm 100 A 150 mm × 2.1 mm ID) was set to 40 °C. The data was evaluated with the LabSolutions program. The LOD and LOQ values were calculated using the Microsoft Excel STEYX function.

Considering that a high number of sample results remained below the LOD and LOQ values, 3 scenarios—lower bound (LB), middle bound (MB) and upper bound (UB)—were used for further calculations. When we estimated the LB scenario, concentration under LOD and LOQ was counted as 0. In case of the MB scenario, concentration under LOD was counted as LOD/2 and concentration under LOQ was counted as LOQ/2. For the UB scenario, concentration under LOD was counted as LOD and concentration under LOQ was counted as LOQ. The actual LOD and LOQ values were 0.031 and 0.093 mg/kg for FB1 and 0.051 and 0.154 mg/kg for FB2, respectively. In each scenario, FB1 and FB2 values were added and considered as a sum to allow comparison with the group TDI established by EFSA. The average mycotoxin concentration was calculated in case of each food raw material and food category.

### 5.2. Food Consumption Data Collection

A Hungarian national food consumption survey was conducted by the National Food Chain Safety Office (NEBIH) between 2018 and 2020, according to the EFSA EU MENU methodology.

Participants were asked to recall information on their food consumption for two non-consecutive days, during personal and telephone interviews, completed with questionnaires. The survey covered all regions of the country, four seasons and all days of the week. Dietary pattern for the whole year was represented, including periodically consumed foods and paying attention to seasonal fruits, vegetables and holiday seasons.

The final database contained 2-day consumption data for 2689 participants (5378 consumption days), including adolescents (10–17 years), adults (18–64 years), the elderly (65–74 years) and other children (below 10 years) as well. The consumption of individual foods was recorded (recipes were disaggregated) in the database, which we screened in the present work for the 11 food categories analysed for mycotoxins.

We arranged the consumption data into two subgroups based on age, in order to account for a more general population and assess the vulnerabilities of the younger generation as well. Therefore, our analysis was conducted for adult consumers (considering all participants above the age of 18) and children consumers (up to 18 years of age). Only consumers who consumed at least one item of the food groups of interest were involved in our calculations, amounting to 5288 consumption days (98.3% of all consumption days), which covered the consumption of 1046 adults and 1598 children.

If a consumer had consumed multiple foods of the same food category on a given day, those consumptions were summed. For this purpose, we grouped the food consumption data into the 11 food categories we analysed during the mycotoxin measurement, as shown in Table 5.

### 5.3. Estimation of Fumonisin Exposure

Fumonisin exposure was estimated by a deterministic method, taking into account the consumption data as a distribution, and the fumonisin concentration (FB1 + FB2 in LB, MB and UB scenarios) data as average values. As the focus of our calculations was chronic intake, for each consumer, the mean daily consumption of each food category was calculated as the average of the two survey days. Then, for each individual consumer, the mean daily consumption of each food category was multiplied by the corresponding average fumonisin concentration (in LB, MB and UB scenarios each); then, the intakes from each food category (*n* = 11) were summed for the specific consumer and divided by their corresponding body weight, which was measured and recorded individually for each consumer. This procedure was repeated for all consumers (1046 adult consumers and 1598 children consumers), and resulted in exposure distributions of both adult and children consumers in all three (LB, MB and UB) scenarios.

The calculation of the daily intake of one consumer can thus be described by using the formula presented in Equation (1), where *EDI* = estimated daily intake (µg/kg BW/day) of one consumer, *A* = amount of all consumed food per day (g/day) in the food category *n*, *C* = average concentration of fumonisin (B1 + B2) in the food category *n* (µg/g), *BW* = body weight (kg) of the specific consumer. This calculation was repeated for each consumer.

(1) $$EDI\;=\;\sum_{n}A\;\times \;C/BW$$

### 5.4. Estimation of Food Category Contributions

The relative contribution of each food category to the total calculated intake was estimated at each selected percentile intake as follows. UB scenarios of child and adult exposures were selected. Each daily total intake was summed up to the percentile of concern (50, 90, 95, 99). Accordingly, intakes from each food category were summed up to the consumption day of the same percentile. The estimated contribution of each food category is the proportion of summed intakes relative to the summed total intake.

The calculations were performed in KNIME software (version 4.7.8) and MS Excel.

## Figures and Tables

**Table 1:** The sample numbers and distribution of FB1 and FB2 concentrations in different food groups.

Food Category	*n*	FB1 < LOD	LOD < FB1 < LOQ	LOQ < FB1	FB1 Mean (mg/kg)	FB1 Median (mg/kg)	FB1 Max (mg/kg)	FB2 < LOD	LOD < FB2 < LOQ	LOQ < FB2	FB2 Mean (mg/kg)	FB2 Median (mg/kg)	FB2 Max (mg/kg)
corn flour	60	14	29	17	0.175	0.162	0.285	45	14	1	0.128	0.128	0.128
cornmeal	60	42	15	3	0.405	0.517	0.59	57	1	2	0.175	0.175	0.185
cornflakes	60	43	13	4	0.063	0.056	0.096	58	1	1	0.027	0.027	0.027
canned corn	60	57	3	0	-	-	-	59	1	0	-	-	-
other corn-based products	73	46	13	14	0.197	0.148	0.531	62	10	1	0.2	0.2	0.2
brown rice	60	58	1	1	0.388	0.388	0.388	59	1	0	-	-	-
white rice	60	59	0	1	0.557	0.557	0.557	59	0	1	0.275	0.275	0.275
rice-based products	60	54	5	1	0.144	0.144	0.144	59	1	0	-	-	-
wheat-based products	59	50	2	7	0.257	0.251	0.587	53	5	1	0.160	0.160	0.160
fine wheat flour	65	65	0	0	-	-	-	65	0	0	-	-	-
whole wheat flour	60	59	1	0	-	-	-	60	0	0	-	-	-

Note: *n* = number of samples, <LOD = number of samples with concentration below LOD (limit of detection), LOQ< = number of samples with concentration above LOQ (limit of quantification). The actual LOD and LOQ values were 0.031 and 0.093 mg/kg for FB1 and 0.051 and 0.154 mg/kg for FB2, respectively.

**Table 2:** The total fumonisin content (FB1 + FB2, mg/kg) of different food groups based on LB, MB and UB scenarios.

Food Category	LB Mean	LB Max	MB Mean	MB Max	UB Mean	UB Max
corn flour	0.052	0.395	**0.115**	0.395	0.178	0.439
cornmeal	0.026	0.775	**0.074**	0.775	0.122	0.775
cornflakes	0.005	0.096	**0.052**	0.173	0.099	0.250
canned corn	0	0	**0.043**	0.124	0.087	0.247
other corn-based products	0.040	0.731	**0.091**	0.731	0.141	0.731
brown rice	0.006	0.388	**0.049**	0.465	0.091	0.542
white rice	0.014	0.832	**0.054**	0.832	0.095	0.832
rice-based products	0.002	0.144	**0.047**	0.170	0.091	0.195
wheat-based products	0.033	0.747	**0.077**	0.747	0.122	0.747
fine wheat flour	0	0	**0.041**	0.041	0.082	0.082
whole wheat flour	0	0	**0.042**	0.072	0.083	0.144

Note: LB = lower bound (<LOQ = 0), MB = middle bound (<LOD = LOD/2, <LOQ = LOQ/2), UB = upper bound (<LOD = LOD, <LOQ = LOQ). The actual LOD and LOQ values were 0.031 and 0.093 mg/kg for FB1 and 0.051 and 0.154 mg/kg for FB2, respectively.

**Table 3:** The estimated exposures of FB1 + FB2 (µg/bwkg/day) in total.

	LB (Adults)	MB (Adults)	UB (Adults)	LB (Children)	MB (Children)	UB (Children)
mean	0.035	0.101	0.168	0.109	0.313	0.516
median	0.028	0.083	0.137	0.086	0.256	0.422
standard deviation	0.034	0.087	0.142	0.095	0.247	0.403
P90	0.075	0.204	0.336	0.219	0.614	1.017
P95	0.096	0.258	0.433	0.280	0.744	1.230
P99	0.144	0.381	0.634	0.444	1.112	1.793
maximum	0.321	0.747	1.173	1.425	3.560	5.694

(Abbreviations: LB, lower bound; MB, middle bound; UB, upper bound; P90, 90% percentile; P95, 95% percentile; P99, 99% percentile).

**Table 4:** Contributions of each food category to the total intake of fumonisins at different percentiles. Results are expressed in %.

Food Category	P50 (Adults)	P90 (Adults)	P95 (Adults)	P99 (Adults)	P50 (Children)	P90 (Children)	P95 (Children)	P99 (Children)
corn flour	0.2	0.1	0.2	0.1	0.0	0.1	0.0	0.0
cornmeal	0.2	0.1	0.1	0.2	0.0	0.0	0.0	0.1
cornflakes	0.4	0.5	0.6	0.5	0.5	0.8	0.7	0.6
canned corn	1.3	2.1	2.8	2.9	1.4	2.5	2.7	3.0
other corn-based products	1.2	0.9	1.0	1.1	4.5	4.8	5.5	5.7
brown rice	0.7	0.5	0.5	0.6	0.2	0.2	0.4	0.4
**white rice**	**17.3**	**15.7**	**15.1**	**14.7**	**13.4**	**14.7**	**14.8**	**14.6**
rice-based products	0.7	0.4	0.4	0.3	1.0	0.9	0.8	0.8
**wheat-based products**	**56.2**	**64.2**	**64.9**	**65.4**	**62.7**	**63.3**	**62.7**	**62.8**
**fine wheat flour**	**21.5**	**15.0**	**14.4**	**13.8**	**16.1**	**12.7**	**12.2**	**11.8**
whole wheat flour	0.4	0.3	0.3	0.2	0.1	0.1	0.1	0.1

**Table 5:** Matching consumption data to the food categories of the present study.

Food Category in the Present Study	Food Consumption Data Obtained from the Survey
corn flour	“corn flour”
cornmeal	“cornmeal”
cornflakes	“cornflakes”
canned corn	“corn, fresh”, “corn, frozen”, “corn, canned”
other corn-based products	“corn chips”, “starch, corn starch”, “corn, for popping”, “corn, popped, without oil”, “corn, popped, with oil”, “corn, popped, cheese flavoured”, “corn, flakes, extruded, natural”, “oil, corn oil”, “puffy, extruded corn flakes, salty”, “nachos tortilla chips”
brown rice	“rice, brown rice”
white rice	“rice, polished”, “rice, unpolished”, “rice, semi-polished”, “flour, rice flour”
rice-based products	“rice chips”, “baby biscuits, organic mini rice cakes”, “cereal porridge, organic natural rice flakes”, “rice, puffed”, “rice, puffed, with paprika”, “rice, puffed, enriched with vitamins and minerals”
wheat-based products	“bread roll”, “bread roll, with grain, rye”, “bread roll, with sesame seeds”, “crescent roll”, “dry pasta”, “dry pasta, gluten-free”, “flat dough”, “puff pastry”, “breadcrumbs”, “yeast dough”, “sweet bread”, “muesli, organic baby muesli”, “muesli, fruit muesli”, “muesli, raspberry-strawberry flavour with banana”, “muesli, organic fruit muesli dessert”, “muesli bar, with chocolate”, “muesli bar, fruity”, “muesli bar, fruity, enriched with calcium”, “salty breadstick”, “choux pastry”, “shortcrust pastry”, “bulgur”, “wheat, germ”, “wheat, semolina”, “wheat, bran”, “seitan”, “hamlet, wheat”, “tortilla, wheat”
fine wheat flour	“flour, wheat flour bl 55”, “flour, wheat flour bl 65”, “flour, durum flour”, “flour, spelt flour”
whole wheat flour	“flour, wheat, whole grain”, “flour, Graham flour bl 96”

## Data Availability

The original contributions presented in this study are included in the article. Further inquiries can be directed to the corresponding authors.

## Abbreviations

The following abbreviations are used in this manuscript:

BW	body weight
DON	deoxynivalenol
EDI	estimated daily intake
EFS	European Food Safety Authority
ELISA	enzyme-linked immunosorbent assay
ESI	electrospray ionisation
FB1	fumonisin B1
FB2	fumonisin B2
FUM	fumonisins
LB	lower bound
LC-MS	liquid chromatography mass spectrometry
LOD	limit of detection
LOQ	limit of quantification
MB	middle bound
NÉBIH	National Food Chain Safety Office of Hungary
TDI	tolerable daily intake
UB	upper bound
ZEN	zearalenone

## Appendix A

**Table A1:** The sample numbers and distribution of FB1 and FB2 concentrations in different food groups, measured during the total time period of 2017–2023.

Food Category	*n*	FB1 < LOD	LOD < FB1 <LOQ	LOQ < FB1	FB1 Mean (mg/kg)	FB1 Median (mg/kg)	FB1 Max (mg/kg)	FB2 < LOD	LOD < FB2 <LOQ	LOQ < FB2	FB2 Mean (mg/kg)	FB2 Median (mg/kg)	FB2 Max (mg/kg)
corn flour	124	26	48	50	0.270	0.181	1.463	84	24	7	0.359	0.342	0.731
cornmeal	122	60	33	29	0.308	0.152	1.959	90	8	6	0.300	0.263	0.577
cornflakes	124	80	32	12	0.127	0.106	0.460	100	2	1	0.027	0.027	0.027
Canned corn	78	74	3	1	0.197	0.197	0.197	73	2	0	-	-	-
other corn-based products	158	94	33	31	0.258	0.161	1.100	110	15	3	0.181	0.172	0.200
brown rice	70	67	2	1	0.388	0.388	0.388	69	1	0	-	-	-
white rice	76	73	2	1	0.557	0.557	0.557	75	0	1	0.275	0.275	0.275
rice-based products	67	61	5	1	0.144	0.144	0.144	66	1	0	-	-	-
wheat-based products	59	50	2	7	0.257	0.251	0.587	53	5	1	0.160	0.160	0.160
fine wheat flour	65	65	0	0	-	-	-	65	0	0	-	-	-
whole wheat flour	61	60	1	0	-	-	-	61	0	0	-	-	-

Note: *n* = number of samples, <LOD = number of samples with concentration below LOD (limit of detection), LOQ< = number of samples with concentration above LOQ (limit of quantification). The actual LOD and LOQ values were 0.031 and 0.093 mg/kg for FB1 and 0.051 and 0.154 mg/kg for FB2, respectively. Some samples were not analysed for FB2 during the 2017–2018 time period.

**Table A2:** The total fumonisin content (FB1 + FB2, mg/kg) of different food groups based on LB, MB and UB scenarios. Mean, median, P95, minimum and maximum values.

Food Category	LB Mean	LB Median	LB P95	LB Min	LB Max	MB Mean	MB Median	MB P95	MB Min	MB Max	UB Mean	UB Median	UB P95	UB Min	UB Max
corn flour	0.052	0	0.275	0	0.395	0.115	0.072	0.352	0.041	0.395	0.178	0.144	0.398	0.082	0.439
cornmeal	0.026	0	0.102	0	0.775	0.074	0.041	0.132	0.041	0.775	0.122	0.082	0.243	0.082	0.775
cornflakes	0.005	0	0.045	0	0.096	0.052	0.041	0.072	0.041	0.173	0.099	0.082	0.144	0.082	0.250
canned corn	0	0	0	0	0	0.043	0.041	0.070	0.041	0.124	0.087	0.082	0.141	0.082	0.247
other corn-based products	0.040	0	0.219	0	0.731	0.091	0.041	0.296	0.041	0.731	0.141	0.082	0.373	0.082	0.731
brown rice	0.006	0	0	0	0.388	0.049	0.041	0.041	0.041	0.465	0.091	0.082	0.082	0.082	0.542
white rice	0.014	0	0	0	0.832	0.054	0.041	0.041	0.041	0.832	0.095	0.082	0.082	0.082	0.832
rice-based products	0.002	0	0	0	0.144	0.047	0.041	0.072	0.041	0.170	0.091	0.082	0.144	0.082	0.195
wheat-based products	0.033	0	0.268	0	0.747	0.077	0.041	0.345	0.041	0.747	0.122	0.082	0.422	0.082	0.747
fine wheat flour	0	0	0	0	0	0.041	0.041	0.041	0.041	0.041	0.082	0.082	0.082	0.082	0.082
whole wheat flour	0	0	0	0	0	0.042	0.041	0.041	0.041	0.072	0.083	0.082	0.082	0.082	0.144

Note: LB = lower bound (<LOQ = 0), MB = middle bound (<LOD = LOD/2, <LOQ = LOQ/2), UB = upper bound (<LOD = LOD, <LOQ = LOQ). The actual LOD and LOQ values were 0.031 and 0.093 mg/kg for FB1 and 0.051 and 0.154 mg/kg for FB2, respectively.

**Table A3:** The summed fumonisin content (FB1 + FB2, mg/kg) of different food groups based on data from 2017 to 2023, LB, MB and UB scenarios.

Food Category	LB Mean	LB Median	LB P95	LB Min	LB Max	MB Mean	MB Median	MB P95	MB Min	MB Max	UB Mean	UB Median	UB P95	UB Min	UB Max
corn flour	0.129	0	0.477	0	2.194	0.182	0.072	0.539	0.0155	2.194	0.236	0.144	0.616	0.031	2.194
cornmeal	0.088	0	0.598	0	2.371	0.132	0.0465	0.610	0.0155	2.371	0.176	0.093	0.621	0.031	2.371
cornflakes	0.012	0	0.108	0	0.460	0.056	0.041	0.142	0.0155	0.537	0.100	0.082	0.168	0.031	0.614
canned corn	0.003	0	0	0	0.197	0.045	0.041	0.072	0.0155	0.274	0.087	0.082	0.144	0.031	0.351
other corn-based products	0.054	0	0.364	0	1.100	0.098	0.041	0.439	0.0155	1.177	0.142	0.082	0.490	0.031	1.254
brown rice	0.006	0	0	0	0.388	0.048	0.041	0.055	0.041	0.465	0.090	0.082	0.110	0.082	0.542
white rice	0.011	0	0	0	0.832	0.052	0.041	0.046	0.041	0.832	0.094	0.082	0.091	0.082	0.832
rice-based products	0.002	0	0	0	0.144	0.046	0.041	0.072	0.041	0.170	0.090	0.082	0.144	0.082	0.195
wheat-based products	0.033	0	0.268	0	0.747	0.077	0.041	0.345	0.041	0.747	0.122	0.082	0.422	0.082	0.747
fine wheat flour	0	0	0	0	0	0.041	0.041	0.041	0.041	0.041	0.082	0.082	0.082	0.082	0.082
whole wheat flour	0	0	0	0	0	0.042	0.041	0.041	0.041	0.072	0.083	0.082	0.082	0.082	0.144

**Table A4:** The estimated exposures of FB1 + FB2 (µg/bw kg/day) considering measurements during the whole time period of 2017–2023.

	LB (Adults)	MB (Adults)	UB (Adults)	LB (Children)	MB (Children)	UB (Children)
mean	0.035	0.101	0.168	0.111	0.313	0.516
median	0.027	0.083	0.137	0.086	0.256	0.421
standard deviation	0.035	0.088	0.142	0.099	0.248	0.403
P90	0.075	0.203	0.336	0.224	0.611	1.016
P95	0.096	0.262	0.437	0.293	0.756	1.230
P99	0.149	0.383	0.647	0.454	1.117	1.793
maximum	0.332	0.747	1.173	1.433	3.564	5.695

(Abbreviations: LB, lower bound; MB, middle bound; UB, upper bound; P90, 90% percentile; P95, 95% percentile; P99, 99% percentile).
